# The microbiome in hematopoietic stem cell transplant recipients and cancer patients: Opportunities for clinical advances that reduce infection

**DOI:** 10.1371/journal.ppat.1006342

**Published:** 2017-06-29

**Authors:** Andrew Y. Koh

**Affiliations:** 1 Department of Pediatrics, University of Texas Southwestern Medical Center, Dallas, Texas, United States of America; 2 Department of Microbiology, University of Texas Southwestern Medical Center, Dallas, Texas, United States of America; 3 Harold C. Simmons Cancer Center, University of Texas Southwestern Medical Center, Dallas, Texas, United States of America; McGill University, CANADA

## Why do cancer and stem cell transplant patients develop invasive bacterial and fungal infections?

In the 1960s, combination chemotherapy (using cytotoxic agents with different mechanisms of action) was adopted to treat cancer patients, resulting in much higher cure rates. During this time, however, a substantial proportion of cancer patients developed bacterial bloodstream infections, predominantly with gram-negative organisms such as *Pseudomonas aeruginosa* and *Escherichia coli*, and the morbidity and mortality associated with these infections was striking. Ironically, these pathogens originated from the patient’s own gastrointestinal (GI) tract, based originally on autopsy results and later using molecular studies [1, 2]. In particular, those patients with severe neutropenia (absolute neutrophil count <100 cells/mm^3^) and fever were found to be at highest risk for developing bacterial and fungal bloodstream infections.

These findings led to sweeping changes in clinical practice whereby initiation of empiric antibiotics for cancer patients with fever and neutropenia became and continues to be standard of care and had a remarkable effect on decreasing the morbidity and mortality associated with these infections [3]. These patients are often admitted for inpatient hospitalization and maintained on antibiotics until the fever has resolved and the neutrophil count has recovered (>500 cells/mm^3^).While treating febrile and neutropenic patients with antibiotics is standard of care, the choice of which antibiotic(s) to use is still largely institution dependent, as a number of antibiotics have been shown to be efficacious in this setting. Thus, cancer and stem cell transplant (SCT) patients often receive multiple antibiotics for long durations of time, and many patients never have a laboratory-documented infection. Cessation of empiric antibiotics before neutrophil recovery, however, is associated with an increased risk of infection [4]. While countless lives have been saved by this practice, clinicians are now facing an increasing number of antibiotic-resistant bacteria in these patients [5]. Furthermore, recent studies have now shown that cancer and SCT patients with significant disruptions in gut microbiota communities are at increased risk for developing invasive infections [2] and the posttransplant complication known as graft-versus-host disease (GVHD) [6–8]. In fact, specific antibiotic therapies, particularly those that destroy specific anaerobic commensal communities, appear to be linked to the development of these complications [5, 8–10]. These data suggest that new, antibiotic-independent approaches to prevent or treat invasive microbial infections in these patients are needed.

## What are the host factors that prevent bacteria and fungi from disseminating from the gut?

The major host immune deficits that promote bacterial and fungal translocation from the GI tract include deficiencies in cellular immunity (particularly neutropenia); impaired intestinal barriers (an adverse consequence of cytotoxic chemotherapy, also referred to as mucositis); and GI microbiota imbalance (often driven by use of broad-spectrum antibiotics). In cancer and SCT patients, as a consequence of cytotoxic chemotherapy, all 3 defense mechanisms are often compromised. In preclinical models emulating the development of gut-derived bacteremia and fungemia, all 3 host defense mechanisms need to be impaired to promote microbial dissemination from the gut: antibiotics to deplete gut commensal microbiota, allowing high levels of pathogenic microbial colonization (i.e., *P*. *aeruginosa* or *Candida albicans*) and cytotoxic chemotherapy (e.g., cyclophosphamide) to both deplete neutrophils and damage GI epithelium [11, 12]. Whereas severe neutropenia and gut mucosal damage are, more or less, experienced by all cancer and SCT patients, there is emerging evidence that those patients with profound disturbances in gut microbiota populations are at higher risk for developing bloodstream infections [2].

## What role does the gut microbiota play in the development of these enteric-derived infections?

**Fig 1:**
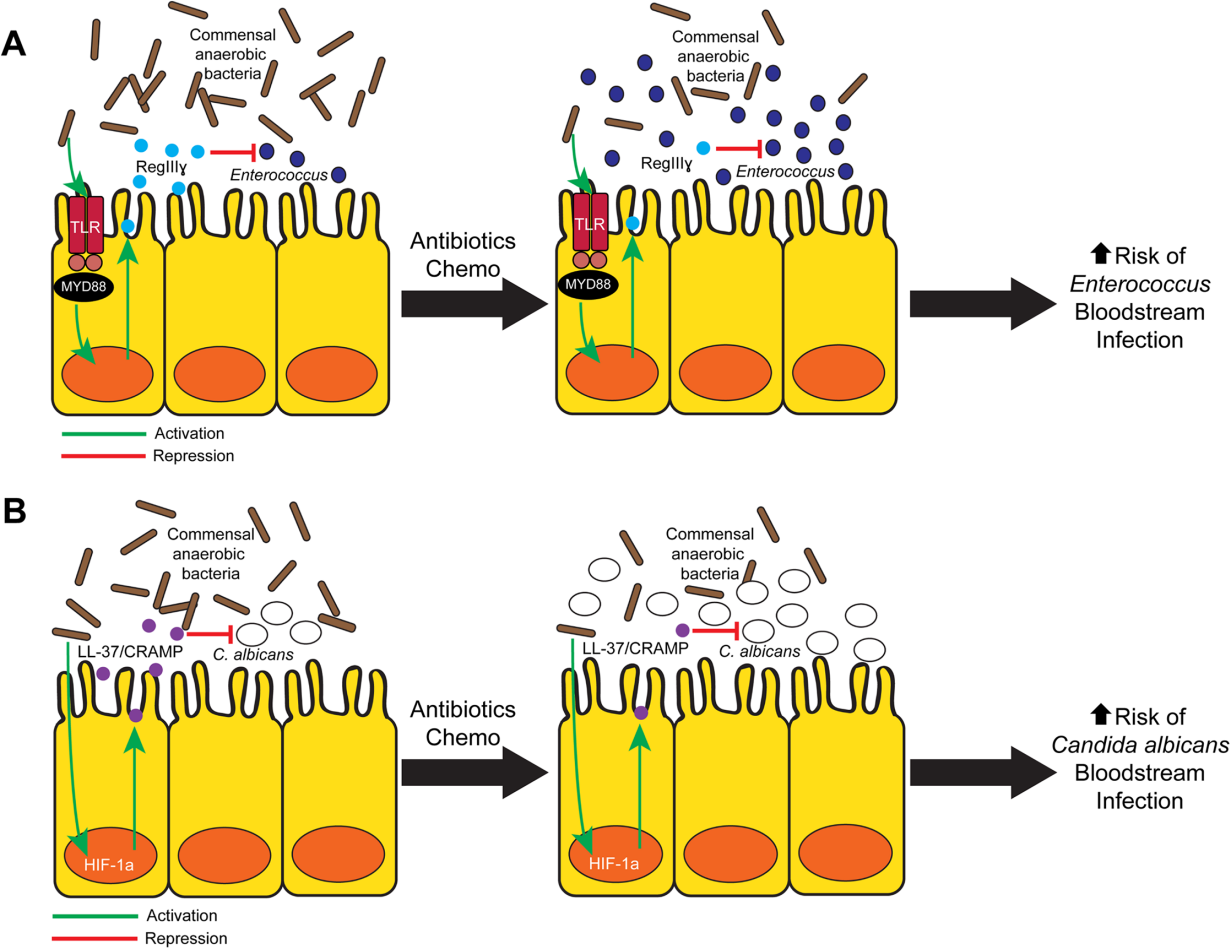
Overview of commensal gut microbiota modulation of colonization resistance to pathogenic bacteria and fungi. **(A)** Commensal gut microbiota induces the intestinal antimicrobial peptide regenerating islet-derived protein 3 gamma (RegIIIγ), which has activity against gram-positive bacteria, including *Enterococcus*, via a TLR/MyD88-dependent mechanism [14]. Antibiotic therapy can deplete commensal gut microbiota, thereby resulting in decreased levels of RegIIIγ and a concomitant increase in *Enterococcus* colonization [17]. Increased gut *Enterococcus* burden is associated with a significantly increased risk of *Enterococcus* bloodstream infections in stem cell transplant patients [2]. **(B)** Commensal gut microbiota (particularly the Bacteroidetes and Clostridial Firmicutes) induce intestinal production of the transcription factor hypoxia-inducible factor-1α (HIF-1α), which in turn regulates production of the antimicrobial peptide LL-37/CRAMP, which has activity against *Candida albicans* [15]. Antibiotic-induced depletion of commensal anaerobic bacteria results in decreased intestinal HIF-1α and LL-37/CRAMP levels and results in increased *C*. *albicans* dissemination in mice [15]. TLR, toll-like receptor; MyD88, myeloid differentiation primary response gene 88.

One beneficial function of the gut microbiota is promoting colonization resistance to pathogens, a process by which the host’s commensal gut bacteria prevent pathogenic bacteria from colonizing the intestine [13]. While the concept of colonization resistance was recognized over 50 years ago, some of the mechanisms underlying colonization resistance have only recently been elucidated. For example, in mice, commensal bacteria can induce GI epithelial cells to produce antimicrobial peptides (AMPs) that are active against bacteria (e.g., regenerating islet-derived protein 3 gamma (RegIIIγ) against *Enterococcus spp*. [14]) (Fig 1A) or fungi (e.g., LL-37/cathelin-related antimicrobial peptide (CRAMP) against *C*. *albicans* [15]) (Fig 1B), thus decreasing GI colonization levels of these potentially pathogenic microbes. When antibiotics are given that deplete commensal microbiota, particularly commensal anaerobes, GI AMP production decreases, pathogenic microbial colonization increases, and the risk of dissemination increases (Fig 1). Not surprisingly, fecal microbiota transplantation or more precise probiotic therapy can restore colonization resistance and thus decrease the risk of infection [15, 16]. Interestingly, even in the setting of antibiotics and depleted levels of commensal microbiota, bacterial ligands (e.g., lipopolysaccharide (LPS) activating toll-like receptor 4 (TLR4) [17]) or pharmacologic agents [15] can be used to induce GI AMP production, reduce pathogenic microbial colonization, and ultimately decrease pathogenic dissemination from the GI tract.

Interestingly, in human SCT patients, an expansion of GI *Enterococcus spp*. or Enterobacteriaceae (along with a concomitant depletion of commensal anaerobic microbiota) is associated with a significantly increased risk of developing bloodstream infection with the same bacterial species [2, 18]. These data suggest that the primary variable that determines which cancer or SCT patients will develop gut-derived microbial bloodstream infections may be those who have significant gut microbiota perturbations, resulting in increased levels or burden of pathogenic bacteria or fungi. Of note, in preclinical models, a 1–2 log–fold reduction in bacterial [17] or fungal gut colonization levels [15] is sufficient to significantly decrease dissemination or mortality. These data suggest that complete eradication or absence of colonization is not needed to achieve a significant decrease in dissemination.

## Should we be monitoring gut microbiota populations in cancer and SCT patients?

With the widespread use of advanced gut microbiota profiling tools, such as 16S rRNA sequencing or metagenomic shotgun sequencing (MSS), the question is whether clinicians should be using real-time monitoring of gut microbiota populations in these patients. For instance, given that increased *E*. *coli* gut microbial burden precedes and significantly increases the risk of *E*. *coli* bacteremia in this patient population [2], frequent monitoring of gut Enterobacteriaceae (the family of bacteria which includes the notable gram-negative pathogens such as *E*. *coli*, *Klebsiella spp*., *Enterobacter spp*., etc.) in cancer and SCT patients could be used to identify those patients at risk for developing Enterobacteriaceae bacteremia.

Unfortunately, both 16S rRNA sequencing and MSS have not been used in the clinical setting because of logistical barriers to implementation—namely, time, cost, and complexity. The turnaround time for 16S rRNA and MSS sequencing data is on the order of weeks to months and thus is far too slow for informing clinical decisions. Another limitation of both 16S rRNA sequencing and MSS is that absolute levels of microbiota cannot be determined, only relative abundance. An increase in gut microbiota relative abundance does not necessarily correlate with an increase in total microbiota levels. In contrast, group or species-specific microbial quantitative polymerase chain reaction (qPCR) performed on patient fecal specimens could theoretically be provided to a clinician within days. qPCR has been applied to 16S rRNA methodology and has been validated for quantification of bacterial groups as well as specific bacterial species within complex bacterial communities [15, 19]. Bacterial species or group qPCR measures the number of gene copies per sample (normalized to both tissue genomic deoxyribonucleic acid (gDNA) concentration and sample weight), not actual bacterial numbers or colony-forming units (CFUs), but qPCR values correlate well with CFU [15, 19, 20]. Furthermore, many clinical microbiology labs already offer numerous pathogen-specific qPCR assays, such as cytomegalovirus, Epstein-Barr virus, adenovirus, etc. In fact, routine screening or monitoring patients for systemic viral infections with qPCR tests (e.g., cytomegalovirus (CMV) qPCR) is standard practice for patients undergoing SCT. Thus, a clinical microbiology laboratory should be able to implement a bacterial qPCR assay for fecal specimens. Bacterial qPCR, however, will not provide a snapshot of the entire gut microbiota population, and thus the clinician will be limited to following levels of the specific bacterial qPCR tests offered by the clinical microbiology laboratory. Therefore, additional studies will need to be conducted to determine which groups/types of bacteria should be followed in order to identify those patients at highest risk for developing invasive microbial infections.

## What does the future hold for the clinical management of invasive microbial infections in cancer and SCT patients?

**Fig 2:**
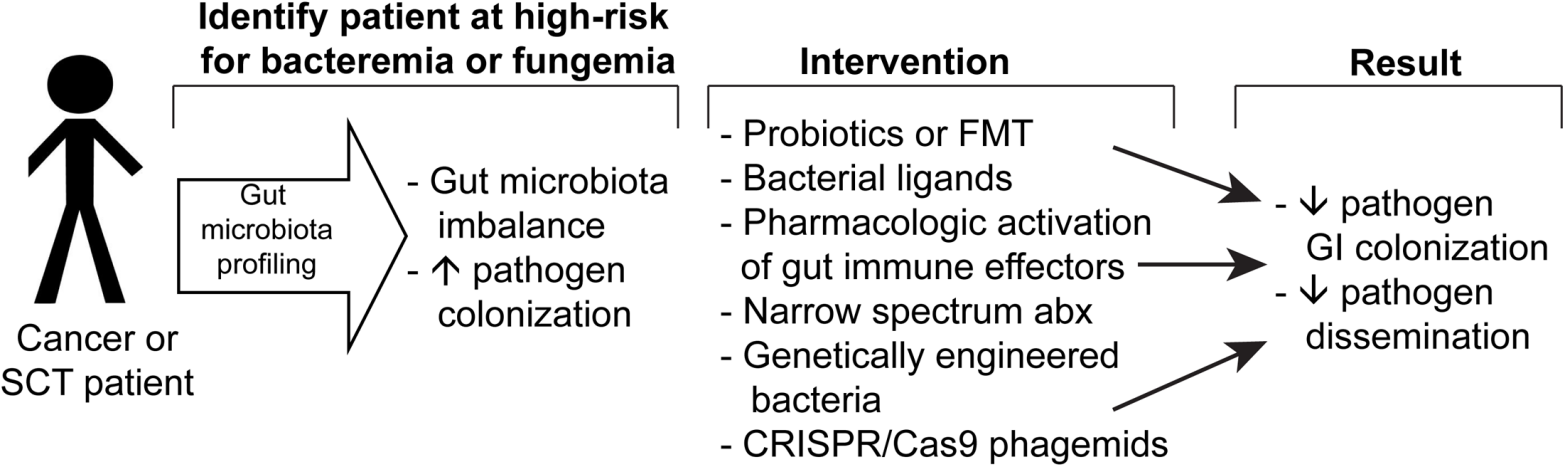
Schema of potential novel approaches to reducing bacterial and fungal infections in cancer and stem cell transplant (SCT) patients. FMT, fecal microbiota transplantation; GI, gastrointestinal.

Prophylactic and empiric antibiotic therapy in cancer and SCT patients has saved countless lives. However, the use of broad-spectrum and possibly unnecessarily prolonged-duration antibiotic therapy has resulted in increasing microbial resistance and unintended deleterious effects to patients as well. In the short term, perhaps more narrow-spectrum antibiotics should be preferentially used to prophylactically treat cancer patients—focusing on covering the gram-negative organisms such as *E*. *coli* and *P*. *aeruginosa*—and relegating broad-spectrum antibiotic use (e.g., meropenem) to when clinical use dictates (e.g., documented extended-spectrum beta-lactamase producing (ESBL) bacteremia). More selective pathogen-target intervention strategies are now being investigated: (1) conjugating an antibiotic to a pathogen-specific antibody [21], (2) genetically engineered bacteria designed to outcompete pathogenic bacteria [22], and (3) clustered regularly interspaced short palindromic repeats/CRISPR associated protein 9 (CRISPR/Cas9) phagemids—plasmids carrying the information to package phage particles that would target specific pathogens [23]. Perhaps, in the future, active monitoring of GI microbiota populations followed by a more targeted manipulation of the gut immune system and gut microbiota populations to either prevent or treat infections or GVHD could become standard of care for cancer and SCT patients (Fig 2).
